# Defining a Conceptual Topography of Word Concreteness: Clustering Properties of Emotion, Sensation, and Magnitude among 750 English Words

**DOI:** 10.3389/fpsyg.2017.01787

**Published:** 2017-10-11

**Authors:** Joshua Troche, Sebastian J. Crutch, Jamie Reilly

**Affiliations:** ^1^Department of Communication Sciences and Disorders, University of Central Florida, Orlando, FL, United States; ^2^Department of Neurodegenerative Disease, Dementia Research Centre, Institute of Neurology, University College London, London, United Kingdom; ^3^Eleanor M. Saffran Center for Cognitive Neuroscience, Temple University, Philadelphia, PA, United States; ^4^Department of Communication Sciences and Disorders, Temple University, Philadelphia, PA, United States

**Keywords:** word concreteness effect, semantic memory, concrete-abstract, lexical-semantic, multidimensional scaling

## Abstract

Cognitive science has a longstanding interest in the ways that people acquire and use abstract vs. concrete words (e.g., truth vs. piano). One dominant theory holds that abstract and concrete words are subserved by two parallel semantic systems. We recently proposed an alternative account of abstract-concrete word representation premised upon a unitary, high dimensional semantic space wherein word meaning is nested. We hypothesize that a range of cognitive and perceptual dimensions (e.g., emotion, time, space, color, size, visual form) bound this space, forming a conceptual topography. Here we report a normative study where we examined the clustering properties of a sample of English words (*N* = 750) spanning a spectrum of concreteness in a continuous manner from highly abstract to highly concrete. Participants (*N* = 328) rated each target word on a range of 14 cognitive dimensions (e.g., color, emotion, valence, polarity, motion, space). The dimensions reduced to three factors: Endogenous factor, Exogenous factor, and Magnitude factor. Concepts were plotted in a unified, multimodal space with concrete and abstract concepts along a continuous continuum. We discuss theoretical implications and practical applications of this dataset. These word norms are freely available for download and use at http://www.reilly-coglab.com/data/.

## Introduction

The word concreteness effect refers to a robust advantage that concrete words manifest over abstract words across numerous domains, including age-of-acquisition, spelling, reading, serial recall, speeded naming, and word recognition (Gilhooly and Logie, 1980; Bleasdale, 1987; Schwanenflugel et al., 1988; Walker and Hulme, 1999; Allen and Hulme, 2006). The origin of the word concreteness effect and its instantiation within the human brain remain among the most contested questions in cognitive neuroscience. Debate over the concreteness effect has accelerated during the past decade, fueled by advances in functional neuroimaging, embodied approaches to modeling the role of emotion in abstract word representation, and computational neuropsychological investigations contrasting the fit of different models within patient-based dissociations (Binder et al., 2005; Crutch and Warrington, 2005; Bonner et al., 2009; Vigliocco et al., 2013).

The Context Availability Model (CAM) proposes the dissociation between abstract and concrete concepts is due to differences in verbal context (Schwanenflugel and Shoben, 1983; Schwanenflugel et al., 1988). In the CAM, verbal context can be understood as information (supplied by discourse or the individual's prior knowledge) that allows for enriched association between concepts. Increased association is postulated to lead to a richer representation of the concept in the brain. Concrete concepts, therefore, have a selective advantage over abstract concepts due to greater availability of contextual information. Stronger availability of contextual information for concrete concepts can be illustrated by comparing a concrete concept such as DOG with an abstract concept such as MAGNITUDE. While associated perceptual information for a concept such as DOG (e.g., leash, bowl) is readily available, analogous relations for a concept such as MAGNITUDE are less available.

Paivio's Dual Coding Theory (DCT) has seen a lengthy and influential tenure as a dominant model of word concreteness (Paivio, 1991). DCT is premised upon the existence of two parallel semantic systems. One component system is dedicated to verbal semantics (i.e., linguistic associations), whereas the other is dedicated to sensory-based feature knowledge (i.e., imaginal semantics). Paivio argues that these systems (or codes) are dissociable but also highly interactive. Concrete words have both sensory and verbal representations and are as such dually coded. In contrast, abstract words inherently lack sensory salience and are, therefore, exclusively coded within the verbal semantic system. The concreteness advantage is conferred from the dual and redundant support of two interactive semantic systems within the human brain.

DCT yields an explicit prediction about the effects of neurological damage on abstract-concrete word representation. That is, damage to the verbal semantic system should produce catastrophic loss of abstract concepts. In contrast, concrete concepts should show resilience to verbal semantic damage due to their redundant representation within a sensory-based semantic code. Conversely, selective damage to the sensory-based semantic code should impact abstract and concrete words uniformly, since both word types share coding within the verbal semantic system. Thus, DCT predicts one type of patient-based dissociation characterized by the loss of abstract words. While a majority of studies display this dissociation (Roeltgen et al., 1983; Coltheart et al., 1987; Katz and Goodglass, 1990; Martin and Saffran, 1992; Franklin et al., 1995) there are a number of studies that demonstrate the opposite dissociation (Warrington, 1975; Warrington and Shallice, 1984; Breedin et al., 1994; Cipolotti and Warrington, 1995; Marshall et al., 1996; Papagno et al., 2007; Reilly et al., 2007) suggesting that the DCT may not be able to completely characterize the representation of concrete and abstract concepts in the brain.

### Alternatives to DCT

The DCT argues that the impoverished representation of abstract concepts has led to differences between abstract and concrete concepts. More recently, two theories have moved from attempting to explain the differences between abstract and concrete concepts in a quantitative manner to a qualitative one. Crutch and Warrington (2005), Crutch and Jackson (2011) argue that abstract and concrete concepts differ based on their relative reliance on association or similarity. They postulate that abstract concepts rely more heavily on associations while concrete concepts rely more heavily on similarity. In other words, a concrete concept such as DOG would rely more heavily on a similar concept such as CAT than an associated concept such as LEASH to support the concept. On the other hand LOVE would rely more on an associated concept such as HATE than a similar concept such as AFFECTION.

Kousta et al. (2011) offer a theory that focuses on the role of affective information. Weak embodiment theories often argue that emotion may play a role in the representation of concepts (Andrews et al., 2009). Kousta and colleagues frame their theory similarly to DCT and argue that concrete concepts are supported by both the verbal and sensorimotor systems. They differ in that Kousta and colleagues argue that abstract concepts are not only supported by verbal systems but also by affective information. They supported this theory by revisiting concreteness effects. (Kousta et al., 2011; Vigliocco et al., 2013) found that when certain psycholinguistic characteristics, such as imageability and context availability, are controlled for, the concreteness effect disappears and even slightly reverses. This finding, however, is not without controversy (see Paivio, 2013).

While we now have a strong theoretical framework to understand the qualitative and quantitative differences between abstract and concrete concepts, it remains an open question as to how these two types of concepts interact in one larger semantic system. We are well able to describe their differences but are less well-equipped to describe their similarities. Crutch and Kousta offer weak embodiment accounts for the representation of abstract and concrete concepts. Embodied theories hold that the brain decomposes objects into discrete sets of features and stores such features within a massively distributed network mirroring perception (e.g., visual features are stored proximal to the visual cortex; Gallese and Lakoff, 2005; Martin, 2007; Barsalou, 2008). Therefore, to begin answering the question of how these concepts interact we must first determine their features. Feature listing approaches have been key to determining the features of concrete concepts (Tyler et al., 2000; Garrard et al., 2001; Cree and McRae, 2003). These approaches often involve participants being given a concept to which they are asked to list its features. This allows for the researcher to determine the discrete features of a concept as well as the common features among categories of concepts. This work has been influential in describing how concrete concepts rely overwhelmingly on sensorimotor information. This work has also helped inform the difference between living and non-living concepts in their representation (Garrard et al., 2001) as well as the primacy of color for the representation of fruit (Cree and McRae, 2003).

There are drawbacks, however, to this approach. While it has shown some utility for abstract concepts (Wiemer-Hastings and Xu, 2005), these feature listing approaches are better suited for concrete concepts. This can be illustrated by trying to determine the features of DRILL as opposed to the features of MAGNITUDE. It was, therefore, necessary to invoke a method that would allow us to determine not just the features of concrete concepts but also abstract concepts. Crutch et al. (2012) piloted a solution to the shortcomings of the feature listing approach in the form of the abstract conceptual feature (ACF) rating approach. This method involved participants rating 50 abstract concepts on a seven point Likert scale on a variety of cognitive dimensions.

### Defining a multi-dimensional semantic space

We previously performed the ACF on 400 nouns (200 abstract & 200 concrete; Troche et al., 2014). This involved participants being asked to rate the 400 nouns on a seven point Likert scale for 12 separate cognitive dimensions. The 12 cognitive dimensions were chosen due to their potential weighting in abstract concept representation (Stuss et al., 1995; Rolls, 2000; Walsh, 2003; Amodio and Frith, 2006; Moscovitch et al., 2006).

We performed an exploratory factor analysis on the data and found that our 12 dimensions could be reduced to three latent factors. These latent factors were characterized as a perceptual salience factor, an affective association factor, and a magnitude factor. We then wanted to determine how these concepts would cluster across these three factors. A hierarchical agglomerative cluster analysis revealed that while there is considerable separation between abstract and concrete concepts, there is also an amount of overlap.

The separation was characterized by differences in perceptual salience. This is not a surprising finding, as by their definition concrete concepts have a perceptual body while abstract concepts do not. A more novel finding was the way affective associations seemed to bind concepts. That is, as concepts increased in affective association they also tended to group together regardless of semantic class. For example LOVE and CHOCOLATE (high affective concepts) were grouped together while INSTANCE and BANANA (low affective concepts) were very far from each other. It was also determined that the Magnitude factor was important for more finely grained distinctions between concepts. This finding can be understood through the concepts of HATE and DISLIKE. While HATE and DISLIKE describe similar feelings, their subtle differences can only truly be described through subtle differences in the scale of these feelings.

We were able to plot the words in a three-dimensional space bounded by the three latent dimensions we created during factor reduction. Plotting the words in this manner allowed us to determine the distance between the concepts using the simple distance metric of Euclidian distance. In effect, we were able to create a semantic space where we could determine how close or far concepts were to each other, with distance in space indicating the relatedness of concepts. We compared the ability of our space to determine the relatedness of abstract concepts to that of Latent Semantic Analysis (Landauer and Dumais, 1997). We found that our space slightly outperformed LSA (Crutch et al., 2013), a highly valid measure of concept relatedness (Howard and Kahana, 1999, 2002; Zaromb et al., 2006) suggesting the validity of the space for abstract concepts.

### Refining a multidimensional semantic space

We identified several problems with the ACF that limited the inference of our initial forays. There were not enough words, we dichotomized concreteness rather than treating it as a continuous variable, and we biased our dimensionality selection in favor of abstract words. In the current paper, we attempted to improve upon the weaknesses of the ACF to create a semantic space that was neurologically valid for all concepts not just abstract concepts and in this way create a method that would allow us to describe the features of both abstract and concrete concepts in a unified manner which would, in turn, allow us to determine not only how concepts differ but also how they relate.

Our current approach, the conceptual feature rating approach (CFR), followed a similar procedure to the ACF. Both approaches asked participants to rate concepts across a variety of dimensions on a seven point Likert scale. The CFR, however, included a greater number of dimensions that would aid in the specificity of the concrete space. We also increased the corpus of words to include not only a greater number of abstract and concrete concepts but also a number of words of middling concreteness which were not included in our previous work. This allowed us to create a continuous semantic space which is closer to the true nature of concepts as opposed to the bimodal space we had previously created.

The greatest difference between the ACF and the CFR approach were the dimensions chosen to be included. We kept the previous dimensions of Emotion, Polarity, Social Interaction, Thought, Morality, Time, Space, and Quantity but also included the dimensions of Visual Form, Auditory, Tactile, Olfactory/Gustatory, Visual Color, and Self-Generated Motion.

### Dimensions

#### Polarity

The overall polarity of concepts (i.e., positive, neutral, negative) was considered as a possible marker of the reward system (e.g., Rolls, 2000) because appraisal of stimulus valence is central to multiple goal-directed behaviors. Valence may be linked to a range of stimulus attributes (e.g., spatial information: up vs. down; large vs. small; emotion: good vs. bad), for example as demonstrated in the space-valence congruence effect (Meier and Robinson, 2004), and may be central to the representation of antonymous semantic relationships (Crutch et al., 2012). In this study, we frame polarity not as the level of positive vs. level of negative emotion but the overall level of emotion regardless of polarity. This was done due to the suggestion by Kousta and colleagues that polarity regardless of poll matters more for processing than whether a concept is positive or negative (Kousta et al., 2009).

#### Emotion

Emotional processing of environmental cues substantially influences our interactions with the world around us (see Dolan and Vuilleumier, 2003) and hence may be expected to also shape our stored knowledge of the world and the language we use to describe it. Some weak embodiment theories have emphasized the contribution to abstract concepts of not only motor and sensory information but also emotion information (e.g., Andrews et al., 2009; Kousta et al., 2009, 2011; Newcombe et al., 2012; see Pecher et al., 2011 for a review). Not all abstract words are affectively loaded, but the acquisition of affectively loaded concepts has been suggested to provide a framework for the subsequent acquisition of non-affective concepts based on linguistic experience alone (Meteyard et al., 2012).

#### Social interaction

Social cognition (labeled as “social interaction” for study participants) was considered because “survival depends upon effective social functioning” (Amodio and Frith, 2006) and such functioning is frequently mediated through verbal communication using a largely abstract vocabulary. Relevant previous work includes the “words as tools” (WAT) proposal that social and linguistic information are particularly important in the acquisition of abstract terms (Borghi et al., 2011; Scorolli et al., 2012), and evidence suggesting the importance of introspection for the development of such concepts (e.g., Barsalou, 1999; Van Overwalle and Baetens, 2009).

#### Morality

Morality was selected as a relevant cognitive dimension in order to capture the association between certain words (e.g., “courage”) and the motivation to act in accordance with certain social or group rules. Moral behavior has been hypothesized to reflect cognitive-emotional association complexes represented across a prefrontal cortex-temporo-limbic network (Moll et al., 2005), and moral concepts have been shown to be particularly vulnerable in patients with frontotemporal dementia (Zahn et al., 2009).

#### Thought

Executive function (labeled as “thought” for the study participants) was selected in an effort to capture the demands that many terms that have multiple meanings or senses in different contexts may place upon skills such as planning, selection, inhibition, executive flexibility, and strategizing (Stuss et al., 1995). It has been suggested that abstract words in particular rely upon computational machinery capable of representing hypothetical physical and mental states, the binding of entities within a structure, and the possible use of embedding (or recursion) in such structures.

#### Time

Time was selected due to its role in the temporal unfolding of event structure (Allman and Meck, 2012). Time is key to our understanding of reality and many disorders lead to disruptions in time judgments that then to lead to cognitive and linguistic deficits (Matell and Meck, 2000; Meck, 2005; Coull et al., 2011).

#### Space

Spatial relationships have been noted to contribute to metaphor (Zwaan and Yaxley, 2003; Lakoff and Johnson, 2008). For many strongly embodied theories of semantic memory, spatial relationships are key to understanding how abstract concepts might achieve sensory grounding. Spatial metaphors are often used to aid in our understanding of abstract concepts such as the saying, “love is a journey” (Gallese and Lakoff, 2005, p. 470). Spatial information has also been discovered to play a role in the way in which concepts are processed. Concepts shown in their iconic spatial relationship (attic above basement) are processed more quickly than concepts shown in a non-iconic spatial relationship (basement above attic; Zwaan and Yaxley, 2003).

#### Quantity

Quantity was included to assess the division between non-numerical and numerical semantics as well as count/mass distinctions (Gathercole, 1985). The difference between the frequency of count nouns and mass nouns are as such; count nouns are nouns that can be modified using a numerical term with no unit of measure required (10 dogs; 15 chairs; 30 pens). Mass nouns cannot be modified using a numerical term unless a unit of measure precedes the noun (10 gallons of water, 15 pounds of sand). While these distinctions seems clear cut, recent work in the literature suggests that this distinction is not as clear and this distinction can shift based on context (Kulkarni et al., 2013). The distinction between these nouns has been found to be important for the acquisition of concepts during development and the conception of concreteness (Gordon, 1985; Chiarelli et al., 2011; Zanini et al., 2016).

#### Visual form

In humans, vision is the dominant sense for interaction with the outside world (Rock and Victor, 1964). It is our most keen sense and a large portion of our brains are dedicated to processing visual information (Mishkin et al., 1983; Van Essen et al., 1992; Drury et al., 1996). Categorization of objects often times only requires input from this sense and feature listing approaches confirm their role in the representation of a variety of concrete concepts (Tyler et al., 2000; Garrard et al., 2001; Cree and McRae, 2003).

#### Auditory

While vision is the most dominant and sensitive human sense, audition remains an important domain as well. The combination of visual and auditory information has been shown to improve object recognition (Stein et al., 1996; Molholm et al., 2004). Also while vision shows primacy for object recognition, audition holds primacy for word recognition and is essential to our access to language.

#### Tactile

Tactile information does not play as large a part in object recognition as auditory and visual form, but in isolation humans can sensitively use tactile information for object recognition (Klatzky et al., 1987; Lederman and Klatzky, 1987). Tactile information has shown importance for the representation of both tools and natural kinds (Chao and Martin, 2000; Tyler et al., 2000; Garrard et al., 2001; Cree and McRae, 2003). It has been suggested that tactile processing and representation may occur in parietal and insular association cortices (Reed et al., 2004)

#### Smell/taste

The chemical senses (taste & smell) were combined in this study. Both senses, in contrast to the visual, auditory and tactile senses, are not represented in the unimodal neocortex but in the limbic and paralimbic cortex (Zatorre et al., 1992; Jones-Gotman and Zatorre, 1993; Small et al., 1999). These brain areas have been associated with affective processing and several studies have shown links between the chemical senses and affective states (Small et al., 1997; Zald et al., 1998; Royet et al., 2000). Olfactory bulb projection also extend to the hippocampus a portion of the brain important for long term memory storage (White, 1965; Scalia and Winans, 1975).

The inclusion of the dimensions Visual form, Auditory, Tactile, & Smell/Taste also fit well within the framework of the influential work by Connell and Lynott (2012; 2013). They determined that the perceptual strength of a concept (i.e., how much a concept is represented by the above dimensions) was better able to predict performance on a lexical decision and word naming task as compared to the concreteness or the imageability of a concept.

#### Color

Color has been found to hold special significance for the representation of fruits and vegetables (Cree and McRae, 2003). It has also been noted, however, that color can aid in the recognition of a variety of objects (Ostergaard and Davidoff, 1985; Wurm et al., 1993; Tanaka and Presnell, 1999). Color is believed to be represented in inferior temporal and posterior parietal cortices (Chao and Martin, 1999).

#### Self-generated motion

Self-Generated Motion has been strongly indicated in the representation of tools (Chao and Martin, 1999; Crutch and Warrington, 2003; Hauk et al., 2004). As with the perceptual features of a concept, the self-generated movements of a tool are acquired through mental simulation. A variety of studies have indicated that the motor cortex plays a role in the representation of action concepts and tools. It has been further shown that concepts show a somatotopic organization in their representation (Farah and McClelland, 1991; Chao and Martin, 2000; Crutch and Warrington, 2003; McRae et al., 2005). In other words, concepts like pencil and hammer would show greater representation in the hand area of the motor cortex while concepts like kick and walk would show greater representation in leg area of the motor cortex.

It should be noted that while we expanded the list of dimensions from our previous work this list of dimensions is by no means exhaustive. Many of these dimensions could be further broken down into a larger set of dimensions. For instance emotion could be broken down further into dominance, valence, and arousal or motion to arm motion and leg motion. We wanted, however, to take care to not overparamitize our model and therefore erred on the side of more coarse dimensions as compared to an exhaustive list of every detailed dimensions. We determined that participants might not appreciate the subtle differences between an exhaustive list which would reduce the uniqueness of each dimension for the model.

## Methods

### Participants

Participants (*N* = 328) were recruited through the online crowd-sourcing program Mechanical Turk. Recent validation studies have shown that the Mechanical Turk data show comparable reliability to standard survey metrics, independent of the task or the amount of payment (Buhrmester et al., 2011). The Mechanical Turk allows users to set specific participant parameters. We isolated participants from the United States who were by self-report native English speakers. Mean age was 34.63 years (range 18–77; SD = 6.45); mean education was 15.03 years (range = 11–20; SD = 1.78). We obtained 328 complete surveys, 173 of which were completed by females. Participants were compensated $6 for ~45 min of work.

### Materials and procedure

Stimuli (*N* = 750) included English nouns drawn from the Medical Research Council (MRC) Psycholinguistic Database (Coltheart, 1981). We eliminated archaic entries and derivatives of other words in the corpus (e.g., bed vs. bedroom). Stimuli were both frequent and familiar as confirmed by the Subtlex American Word Frequency norms (Brysbaert and New, 2009) and the MRC familiarity norms (Coltheart, 1981). Average word frequency was 50.7 per-million words (SD = 160.97, range = 0.02–2759.2). Average word familiarity was 526 on a 100–700 point scale (SD = 55, range = 334–646).

Our aim was to obtain a diverse sample of words varied in concreteness from highly abstract to highly concrete, including a neutral subset of stimuli. The MRC Psycholinguistic database word concreteness ratings reflect an amalgamation of three smaller datasets (Paivio et al., 1968; Toglia and Battig, 1978; Gilhooly and Logie, 1980). These datasets were subsequently rescaled to a common 100–700 metric with a mean of 438 and a standard deviation of 120. We sampled a continuous spectrum of concreteness, ranging from highly abstract words (e.g., assumption) to highly concrete (e.g., tomato). A key aspect of this sampling procedure is that we also included words in the middling range concreteness (e.g., damage). One significant advantage of this sampling procedure is that it treats concreteness as a continuous, rather than dichotomous variable.

### Cognitive dimensions and scale instructions

The 750 words were rated across 14 cognitive dimensions by participants. Participants signaled their rating for each word via mouse click in a horizontally arrayed series of survey bubbles, corresponding to a seven-point Likert Scale. This scale ranged from Strongly Disagree (1), through Neutral (4), to Strongly Agree (7). The specific wording we used for rating each cognitive dimension was as follows:

*Polarity* “I relate this word to positive or negative feelings in myself.”
*Thought* “I relate this word to mental activity, ideas, opinions, and judgments.”
*Emotion* “I relate this word with human emotion.”
*Social Interaction* “I relate this word with relationships between people.”
*Time* “I relate this word with time, order, or duration.”
*Space* “I relate this word to position, place or direction.”
*Quantity* “I relate this word to size, amount or scope.”
*Morality* “I relate this word to morality, rules or any other thing that governs my behavior”
*Visual form* “I relate this word to shapes, forms, textures that I can see with my eyes.”
*Tactile* “ I relate this word to sensations (e.g., texture, shape, temperature, wetness) I can feel with my hands or body.”
*Smell/Taste* “I relate this word to flavors and odors I can smell and/or taste.”
*Auditory* “I relate this word to sounds, rhythms, etc. that I can hear.”
*Color* “I relate this word to color.”
*Self-Motion* “I relate this word to my own self-generated movement.”

Participants were instructed to use the entire scale and to work quickly but carefully. The order of presentation both between and within the cognitive dimensions was fully randomized.

### Data analysis procedures

We first pursued dimension reduction via factor analysis with a Varimax Rotation and Kaiser Normalization (Kaiser, 1958) using SPSS-21. We extracted factors with Eigenvalues >1 and generated orthogonalized factor scores using the Anderson and Rubin (1956) method. We then submitted the factor scores for each word (*N* = 750) to a hierarchical agglomerative cluster analysis. This clustering algorithm involves a bottom-up approach, aggregating clusters upward until attaining a stopping point. We used Ward's method of clustering (Ward, 1963) and determined the optimal clustering solution by comparing partitional k-means solutions using the Cohen's Kappa statistic as per the method proposed by Aldenderfer and Blashfield (1984).

## Results

### Inter-rater reliability

We determined the inter-rater reliability of each dimension by examining the two-way mixed intraclass correlation (ICC). All dimensions had excellent inter-rater reliabilities (Table 1) according to Cicchetti's (1994) guidelines.

**Table 1:** Inter-rater reliability across dimensions.

**Dimension**	**ICC**
Emotion	0.89
Polarity	0.90
Social Interaction	0.81
Morality	0.91
Motion-Self	0.82
Thought	0.82
Color	0.85
Olfactory-Gustatory	0.90
Tactile	0.84
Visual Form	0.94
Auditory	0.88
Space	0.84
Quantity	0.82
Time	0.84

*ICC, Intraclass Correlation*.

### Attributes of the individual predictors prior to clustering

**Figure 1:**
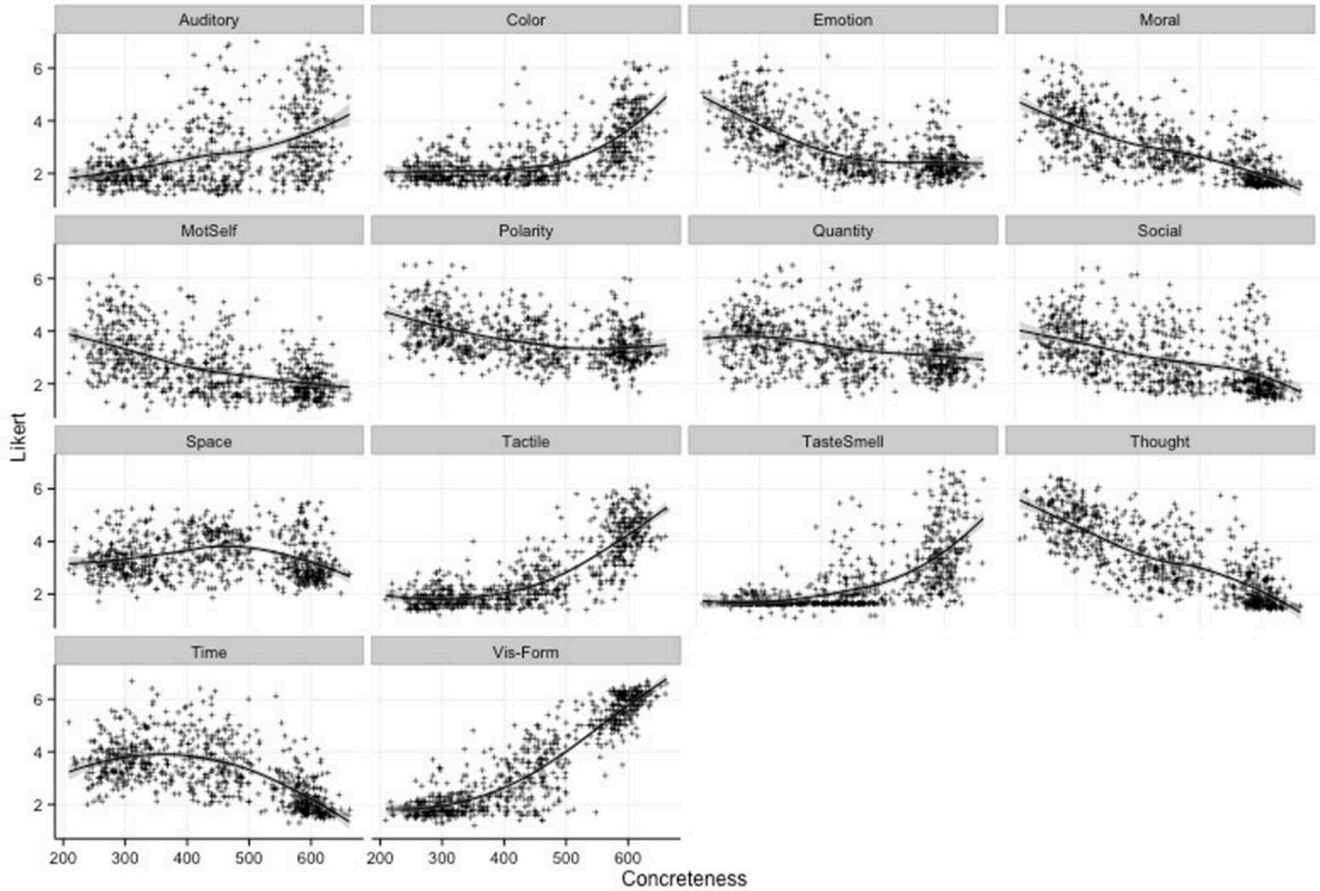
Scatterplots of word concreteness by dimension with fit line included. The x-axis represents the concreteness of the concept while the y-axis represents the mean likert score of each concept for each domain. Curves were fitted using the Loess Method as implemented within the R statistical base package. Mot-Self, Self-Generated Motion; Social, Social Interaction; Vis-Form, Visual Form.

**Figure 2:**
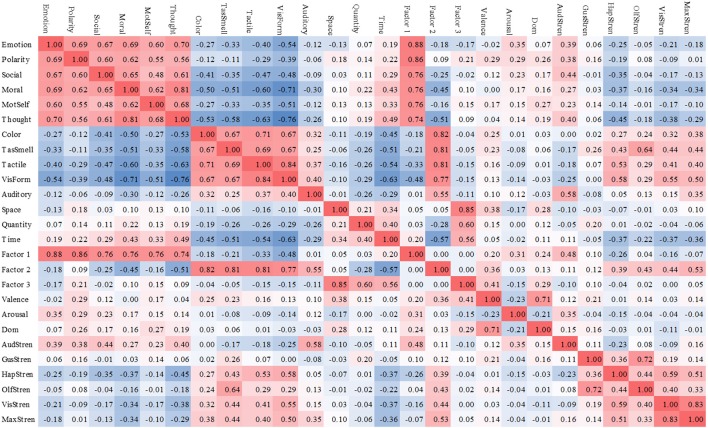
Correlations between dimensions, factor scores, Wariner et al. norms, and Connell & Lynott norms. Social, Social Interaction; MotSelf, Self-Generated Motion; TasSmell, Taste/Smell; VisForm, Visual Form; Dom, Dominance; AudStren, Auditory Strength; GusStren; Gustatory Strength; HapStren; Hapatic Strength; OlfStren, Olfactory Strength; VisStren, Visual Strength; MaxStren, Maximum Perceptual Strength (maximum modality strength rating).

Figure 1 represents scatterplots of the original 14 cognitive dimensions reflecting salience of each domain (x-axis) relative to its respective Likert scale rating (y-axis). Figure 2 represents a correlation matrix describing bivariate correlations between each of the individual predictors and factor scores with the Perceptual Strength Norms (Lynott and Connell, 2013) and the Affective Rating Norms (Warriner et al., 2013).

### Factor analysis

Using an Eigenvector >1 extraction criterion, SPSS-21 extracted three latent factors from the original 14 dimensions. Table 2 reflects the component matrix.

**Table 2:** Component matrix.

	**Principal component**		
**Dimension**	**Factor 1**	**Factor 2**	**Factor 3**
Emotion	0.88		
Polarity	0.86		
Social Interaction	0.76		
Morality	0.76		
Motion-Self	0.76		
Thought	0.74		
Color		0.82	
Olfactory-Gustatory		0.81	
Tactile		0.81	
Visual Form		0.77	
Auditory		0.55	
Space			0.85
Quantity			0.60
Time			0.56

Factors aggregated as follows:

- Factor 1 (Endogenous): Emotion, Polarity, Social Interaction, Morality, Motion Self-Generated, Thought
- Factor 2 (Exogenous): Color, Olfactory/Gustatory, Tactile, Visual Form, Auditory
- Factor 3 (Exogenous): Space, Quantity, Time

**Figure 3:**
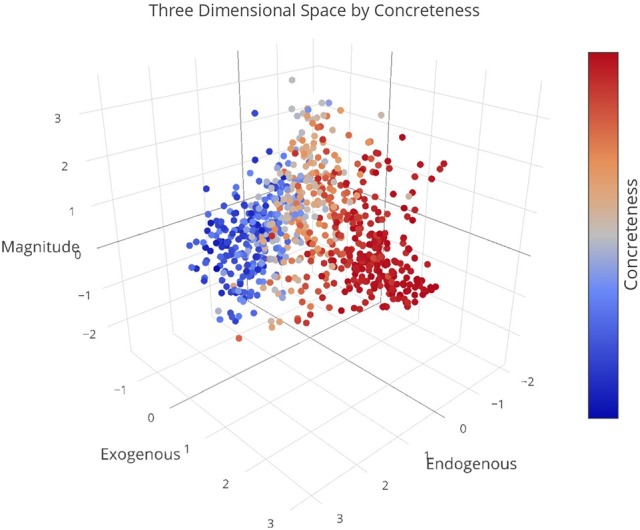
Three-dimensional semantic space with the color of the dots representing concreteness. Redder colors represent more concrete concepts while bluer colors represent more abstract concepts. The x, y, and z-axis represent the endogenous, exogenous, and magnitude scores respectively of each concept.

**Figure 4:**
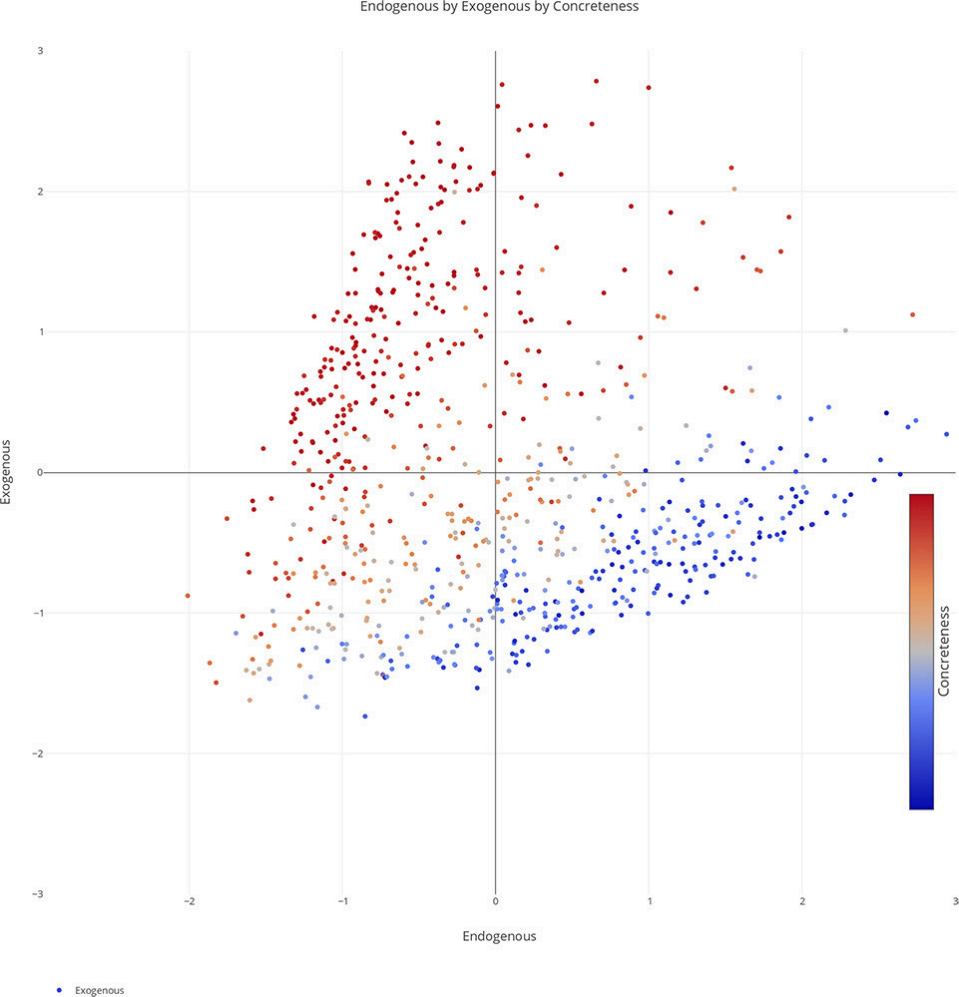
Two-dimensional scatterplot of semantic space with the color of the dots representing concreteness. Redder colors represent more concrete concepts while bluer colors represent more abstract concepts. The x, y-axis represent the endogenous & exogenous scores respectively of each concept.

**Figure 5:**
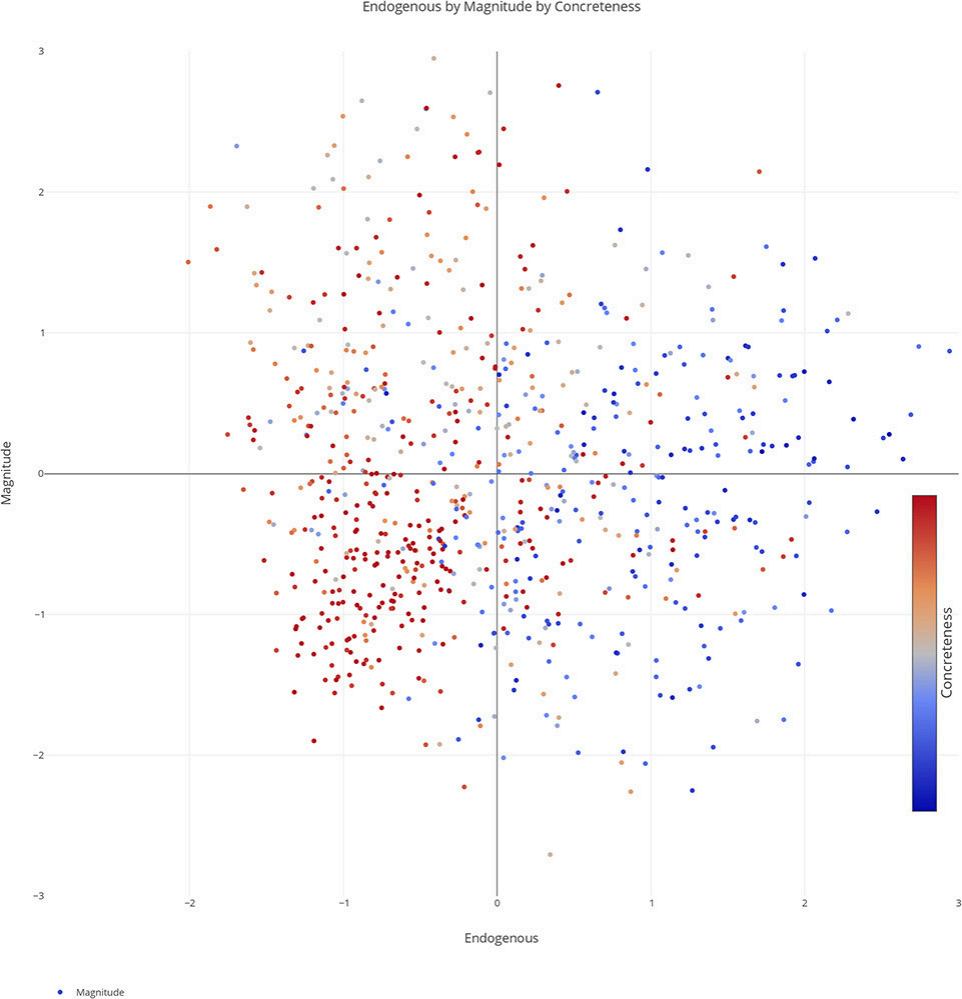
Two-dimensional scatterplot of semantic space with the color of the dots representing concreteness. Redder colors represent more concrete concepts while bluer colors represent more abstract concepts. The x, y-axis represent the endogenous & magnitude scores respectively of each concept.

**Figure 6:**
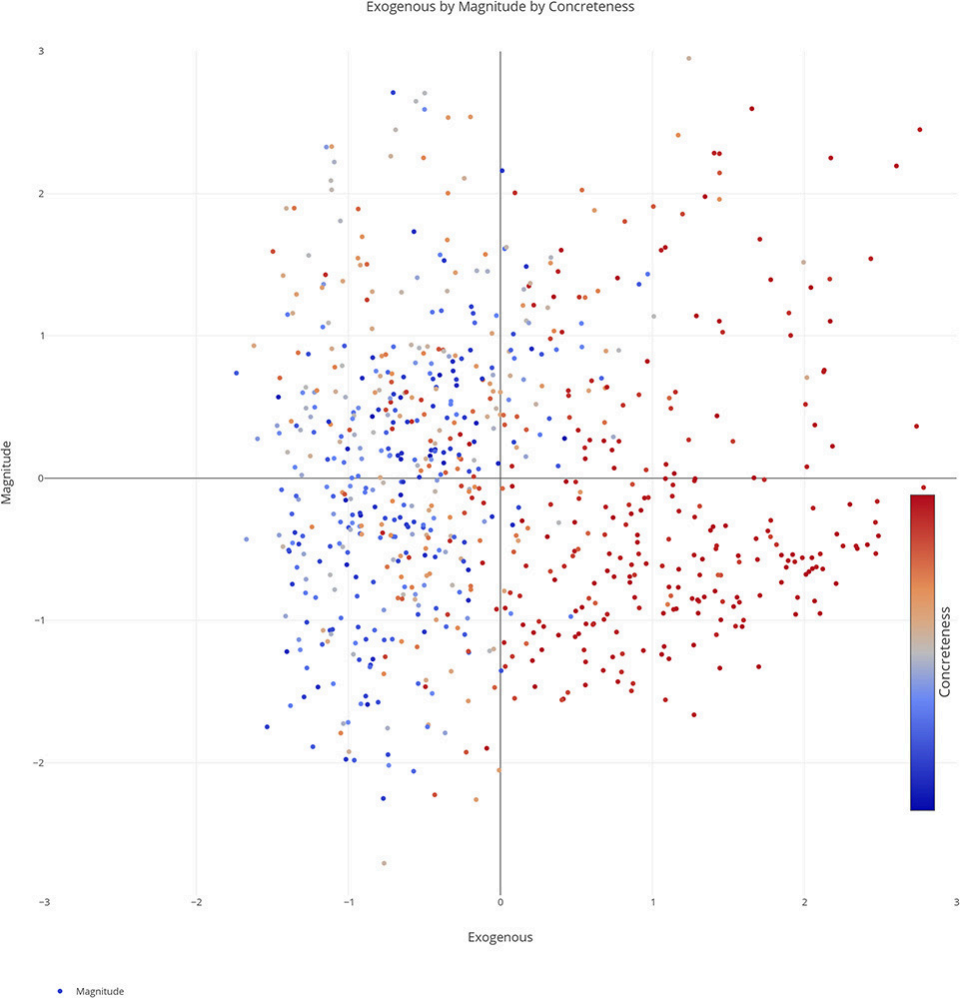
Two-dimensional scatterplot of semantic space with the color of the dots representing concreteness. Redder colors represent more concrete concepts while bluer colors represent more abstract concepts. The x, y-axis represent the exogenous & magnitude scores respectively of each concept.

**Figure 7:**
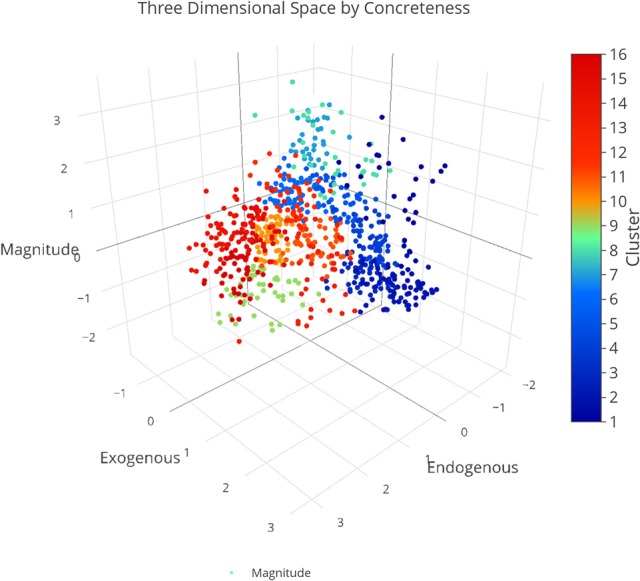
Three-dimensional semantic space with the color of the dots representing cluster membership. Each individual color represents a separate cluster from the cluster analysis. The x, y, and z-axis represent the endogenous, exogenous, and magnitude scores respectively of each concept.

**Figure 8:**
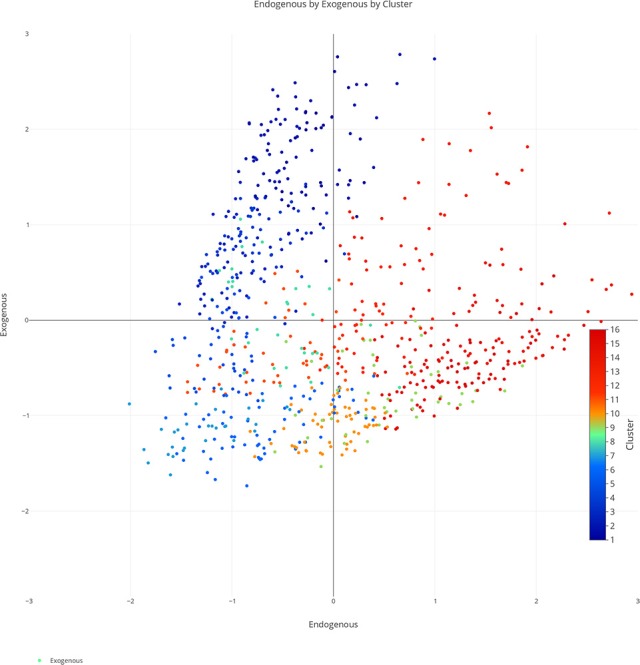
Two-dimensional semantic space with the color of the dots representing cluster membership. Each individual color represents a separate cluster from the cluster analysis. The x, y-axis represent the endogenous & exogenous scores respectively of each concept.

**Figure 9:**
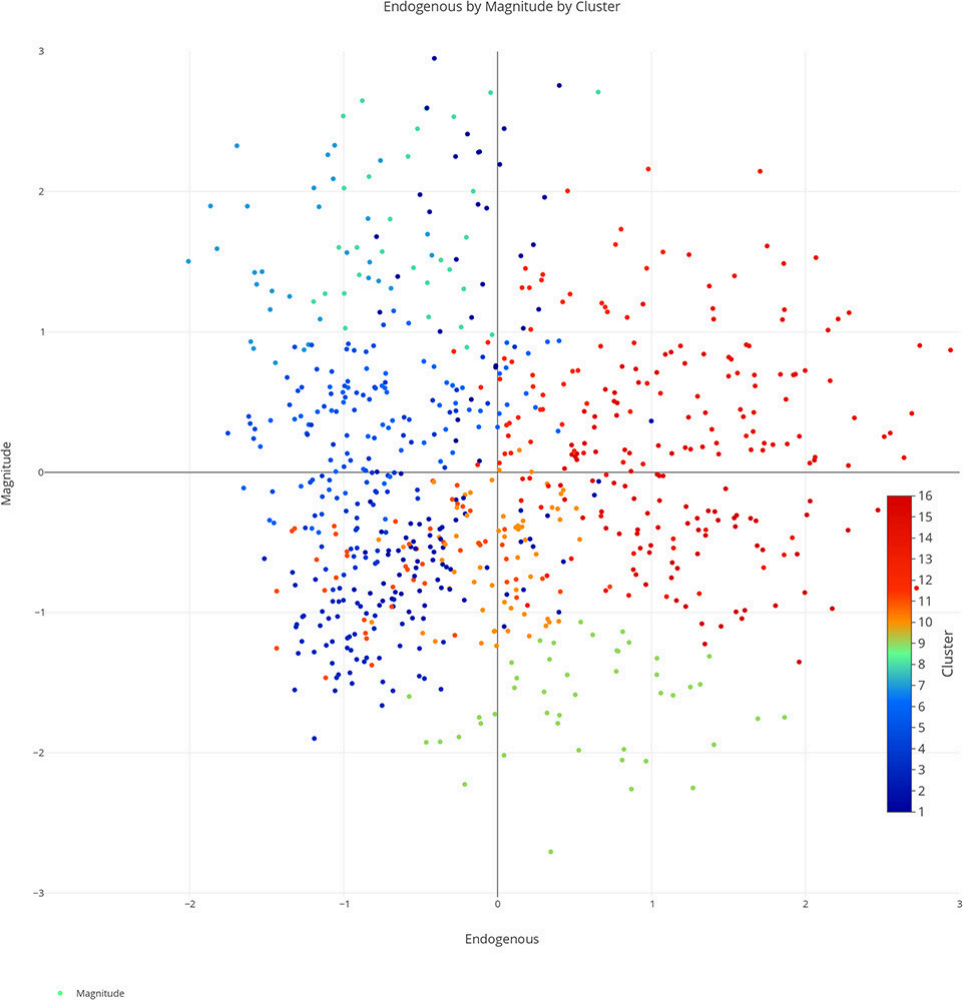
Two-dimensional semantic space with the color of the dots representing cluster membership. Each individual color represents a separate cluster from the cluster analysis. The x, y-axis represent the endogenous & magnitude scores respectively of each concept.

**Figure 10:**
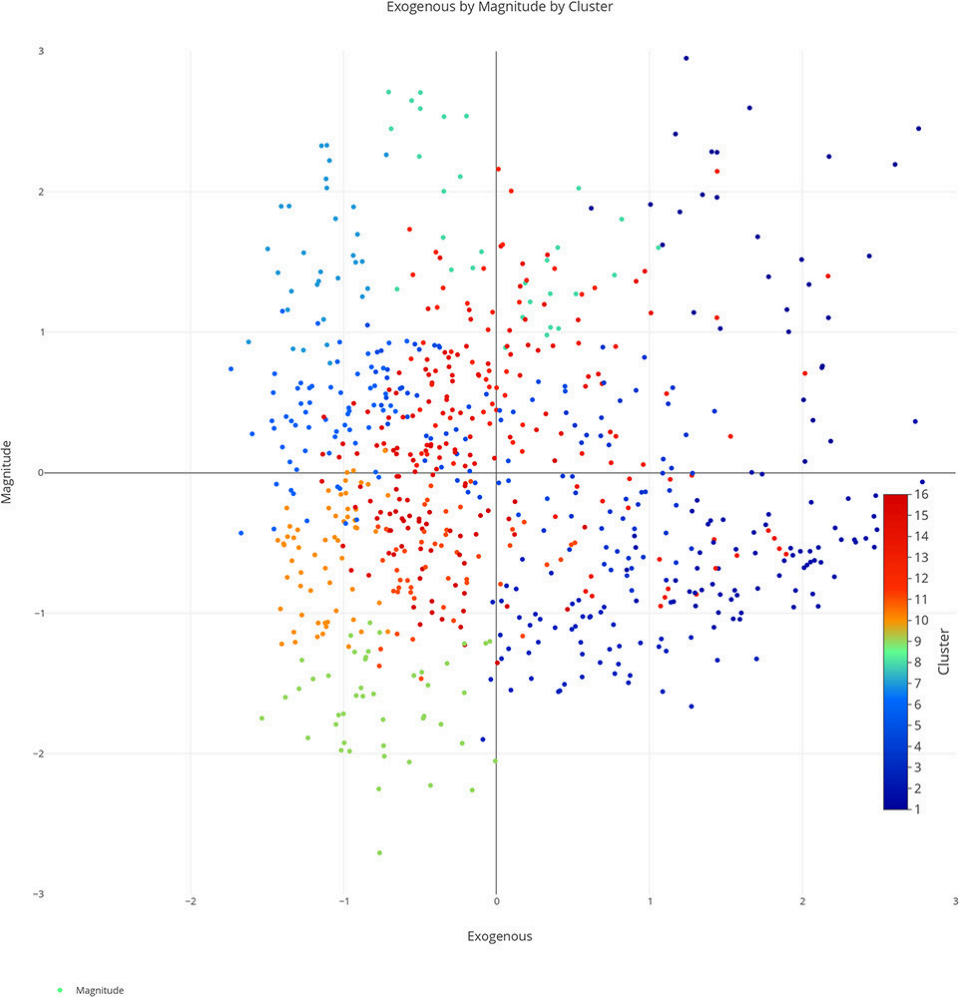
Two-dimensional semantic space with the color of the dots representing cluster membership. Each individual color represents a separate cluster from the cluster analysis. The x, y-axis represent the exogenous & magnitude scores respectively of each concept.

We plotted the factor scores in order to create a visual representation of the semantic space. We created a three-dimensional scatterplot of the space along with two-dimensional scatterplots as it can be difficult to determine patterns in a three dimensional plan. We created two versions of each plot, one set of plots was colored based on the concreteness of the concepts (Figures 3–6) and the other was based on the cluster membership which is described below (Figures 7–10).

### Hierarchical cluster analysis

**Figure 11:**
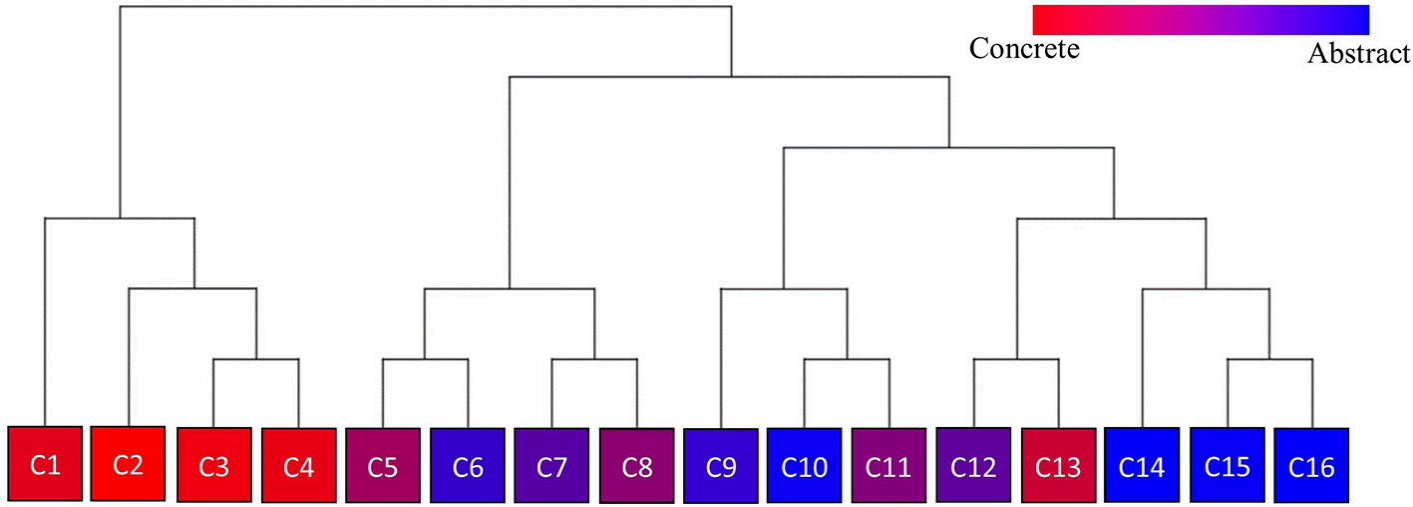
Hierarchical cluster analysis with cluster numbers. Color of the cluster is representative of the concreteness of the words inside of the cluster.

We evaluated various cluster sizes using Cohen's Kappa, and a 16-cluster solution (Kappa = 0.89) best fit the data. We then conducted a hierarchical cluster analysis, starting with 16 clusters, agglomerating upward through six distinct levels until achieving convergence. Figure 11 is a visual representation of our cluster analysis which also illustrates the concreteness of the nouns inside of the cluster. Table 3 reflects the mean dimension rating for the nouns inside of the clusters.

**Table 3:** Clustering properties for the 750 english target words.

	**C1**	**C2**	**C3**	**C4**	**C5**	**C6**	**C7**	**C8**	**C9**	**C10**	**C11**	**C12**	**C13**	**C14**	**C15**	**C16**
Polarity	3.9	3.5	2.9	3.2	3.0	3.3	3.1	3.7	3.8	3.6	3.2	4.1	4.5	5.2	4.2	4.5
Smell-Taste	4.2	4.7	3.0	3.2	2.4	1.7	1.7	2.4	1.8	1.6	2.2	2.7	3.2	1.8	1.8	1.7
Visual Form	5.4	6.0	5.8	5.7	4.3	2.4	2.7	3.7	2.5	2.0	3.7	2.9	4.7	2.1	1.9	2.0
Tactile	4.1	4.7	4.3	4.3	2.8	1.8	1.9	2.7	1.9	1.7	2.5	2.1	3.6	2.0	1.9	1.9
Auditory	4.0	3.8	3.3	3.2	2.3	1.9	1.6	2.5	2.6	2.0	3.2	3.1	4.1	2.4	2.1	2.6
Color	4.2	4.5	3.3	3.4	2.4	1.9	1.9	2.6	1.9	2.0	2.3	2.3	3.0	2.2	2.0	2.2
MotionSelf	2.3	2.0	1.8	2.0	1.8	2.3	2.0	2.4	2.6	2.7	2.2	3.2	2.9	4.3	3.6	4.0
Morality	2.1	1.8	1.8	1.9	2.3	2.9	2.6	2.7	3.6	3.5	2.5	3.2	2.9	4.8	4.0	4.2
Emotion	2.4	2.4	2.2	2.3	2.0	2.6	2.1	2.4	4.2	3.3	2.5	2.9	3.6	4.7	3.9	5.0
Space	4.6	2.8	2.6	3.5	3.9	3.5	4.1	4.6	2.5	2.8	3.1	4.4	3.5	3.9	3.4	3.1
Quantity	3.9	2.7	2.6	3.2	3.5	4.0	4.9	4.5	2.6	3.6	2.7	3.1	2.9	4.0	3.7	3.2
Social	2.2	2.0	1.9	2.1	2.2	2.8	2.2	2.5	3.7	3.2	2.9	3.3	4.2	4.3	3.8	4.2
Time	2.8	1.9	2.0	2.3	3.2	4.2	4.8	4.1	2.7	3.5	2.9	3.9	3.0	3.9	3.8	3.3
Thought	2.2	1.8	2.0	2.1	2.6	3.6	2.7	2.9	4.1	4.1	3.0	4.2	3.4	5.4	4.7	5.1

*Dimension mean for the words encapsulated in each cluster*.

## Discussion

In this experiment we applied the Conceptual Feature Rating (CFR) approach as a method to describe the features of both abstract and concrete concepts in a unified space. We improved upon our initial method by not only including dimensions from our previous work but also by expanding our approach to other dimensions suggested by feature listing approaches and the work on perceptual strength by Connel and Lyott (Connell and Lynott, 2012; Lynott and Connell, 2013) as holding significance for the representation of concrete concepts. We also included concepts from across the whole concreteness spectrum not just those at the extremes of the spectrum.

### Factor analysis

As with our previous work, the dimensions reduced to three latent variables. These three variables, however, had some nuanced differences. In our previous work, we described the latent variables as encompassing a sensorimotor variable, a social/affective information variable, and a magnitude variable. In this work the three latent variables represented an Endogenous variable, an Exogenous variable, and a Magnitude variable. The Endogenous variable contained the dimensions of Emotion, Polarity, Social Interaction, Morality, Self-Generated Motion, and Thought. The Exogenous variable contained the dimensions of Color, Olfactory/Gustatory, Tactile, Visual Form, and Auditory. Finally the Magnitude variable contained Space, Quantity, Time. Overall the Exogenous and Endogenous variables were similar to the variables in our previous work.

The Magnitude variable had a slight difference in this study as compared to our previous work. The factor reduction for our ACF space reduced the dimensions of Space and Quantity into one latent variable which we classified as Magnitude while the Time dimension reduced into what we are describing in this study as the Exogenous variable. For our current factor reduction, not only Space and Quantity reduced into a latent variable but Time as well. We mused in our other work why Time had not reduced with Space and Quantity and the current reduction is more in line with what Walsh (2003) described as a magnitude system. These two findings, however, and mixed and likely require further study.

The Endogenous variable contained, as with our social/affective information variable from our past work, the dimensions of Emotion, Polarity, Morality, Thought, Social Interaction. It also contained, however, the dimension of Self-Generated Motion. This dimension was not present during the ACF analyses. The fact that this dimension reduced with these other dimensions lead us to conclude that this variable may be more than just a variable of social/affective information. This variable may be more indicative of a difference between information which arises from the Self vs. information which arises from the outside (Endogenous vs. Exogenous).

### Correlation

Figure 2 presents both the uniqueness of our findings but also its relation to previous work. This figure is a bivariate correlation matrix of our dimension and factor scores with the Perceptual Strength Norms (Lynott and Connell, 2013) and Affective Rating Norms (Warriner et al., 2013). A closer examination of the correlations between the factor scores with these norms reveals several interesting findings. Factor 1 our Endogenous variable has significant correlations with Arousal (0.31) and Auditory Strength (0.48). Factor 2 the Exogenous variable has strong correlations with many of the perceptual strength measures (Haptic Strength = 0.39; Olfactory Strength = 0.43; Visual Strength = 0.44; Max Perceptual Strength = 0.53). Factor 3 the Magnitude variable displayed significant correlations with Valence (0.41) and Dominance (0.29) suggesting that the Magnitude variable does indeed play a role in teasing apart the subtle differences across affective concepts.

### Cluster analysis

Our cluster analysis revealed both an improvement in our overall method from our previous work but also some important theoretical findings. Through the inclusion of greater dimensionality, we were able to improve the specificity of our space for concrete concepts. Figure 11 is a visualization of our hierarchical cluster analysis. The left side of the cluster analysis (C1–C4) contains mostly concrete concepts. In our ACF approach concrete concepts clustered into large undifferentiated clusters. In this approach concrete concepts grouped across four smaller clusters which were differentiated across categories such as landscapes (e.g., FOREST, LAKE, VOLCANO; C1), natural kinds (CATTERPILLER, LIZARD, PIGEON; C2) and tools (e.g., KNIFE, CUP, LAMP; C3). Overall these findings display that this approach can be used to specify the features of both abstract and concrete concepts with a common method.

Further inspection of the cluster analysis revealed trends that were similar to those seen in the semantic space created by the ACF. Globally, abstract and concrete concepts split. Concrete concepts were more dominant on one side of the cluster analysis while abstract concepts were more dominant on the opposite side. It should also be noted that concrete concepts had higher Exogenous variable ratings while abstract concepts had higher Endogenous variable ratings overall.

A closer inspection revealed, as with previous work, that the Endogenous variable had the ability to draw concepts high in the Exogenous variable toward the right side of the cluster analysis (see Figures 7–10). In other words, concepts like DAD and BABY (C13), even though firmly concrete, were considered related to concepts like LOVE and TRUST (C14). These findings fit well with the assertions of other researchers that Endogenous information such as Emotion, Polarity, Social Interaction can change the representation of a concept, even a concrete concept (Altarriba et al., 1999; Andrews et al., 2009; Kousta et al., 2011).

The Magnitude variable had similar effects on the clustering of concepts as in the ACF. The magnitude variable, while not leading to great shifts in the clustering of concepts as with the Endogenous and Exogenous variable, did play a key role in the fine-grained discrimination of concepts. We have previously argued that a Magnitude system is necessary in the semantic system in order to differentiate between concepts such as HATE and DISLIKE. It also was important in characterizing the differences between concepts of distance (e.g., MILEAGE, INCH, LENGTH; C7) and other abstract scientific conceptualizations (e.g., CONCLUSION, CALCULATION, DISTRIBUTION; C6).

We also observed that even though polarity (at least not as described by Bradley and Lang) and dominance were not directly measured, items did cluster across these variables. Positive emotions such as LOVE, FREEDOM, and HOPE (C14) clustered separately from Negative emotions such as GRIEF, SADNESS, and MISERY (C16). Concepts low in dominance such as HELPLESSNESS, FEAR, and GUILT (C9) clustered as well.

### Implications

These findings have interesting implications for how we understand the relationship between abstract and concrete concepts. Our CFR space not only allows us to describe the features of just abstract and just concrete concepts but also to describe the relationship between abstract and concrete concepts. By offering a unimodal, multidimensional semantic space, we are able to describe concepts in a more naturalistic manner.

There remains much controversy on the nature of concreteness in the literature. A large body of work suggests that abstract and concrete concepts are separate and unique entities with a bimodal distribution (Brysbaert et al., 2014). There is a growing body of literature, however, that argues that concreteness is a continuous spectrum (Kousta et al., 2009, 2011; Vigliocco et al., 2013). This work suggests that abstract and concrete concepts can be modeled on a continuous spectrum and that a multimodal approach can not only describe the similarities but the differences between the concepts. Inspection of the three dimensional semantic space does reveal that concepts position in space is highly influence by sensory states (i.e., how imageable a concept is), but it also reveals that this difference is graded, not bimodally as seen in some portions of the literature. The inclusion of endogenous information such as emotion and the magnitude information led to a continuous and graded spread of the concepts. It reveals that while the concreteness of a concept is important for its representation, endogenous information and magnitude information also play an important role in the way a concept is represented. We see that endogenous information can bind concepts regardless of their concreteness which affirms more recent work on the relationship between emotion and concreteness (Kousta et al., 2011; Vigliocco et al., 2013). We also see, more novelly, that magnitude information can play an important role in the separation of concepts at the microlevel (i.e., HATE vs. DISLIKE). It should be noted that this work in no way denies the importance of the abstract and concrete distinction. What we argue is that the distinction plays an important part in the representation of concepts along with endogenous information and magnitude information and that these factors can be modeled in a unified, multidimensional space creating a continuous concreteness spectrum.

### Validity

The stability of these results across this study and our previous study were a strong indication of the validity of this approach, but we have also begun to test the validity of this space using formal behavioral testing. As previously discussed Crutch and colleagues have determined that abstract and concrete concepts rely differentially on similarity or associative information. Garcia et al. (2015) found that the concepts in the CFR space were able to model the similarity and association differences between concepts effectively.

The ability to model both similarity and association may have been a factor in why the CFR model outperformed Latent Semantic Analysis (Landauer and Dumais, 1997) at determining the relatedness between concepts in a word relatedness task. A triad of words was shown to participants in which they had to determine which of the bottom two words most matched the top word (Troche and Reilly, 2016). In these tasks, the more related the target and probe are the more quickly, and easily the relationship is discovered. Items that were highly related in the CFR space as compared to the LSA space were found to be more quickly and easily processed. These findings, due to the validity of the LSA space, are a strong indication of the validity of both CFR space and the method used to create the space.

### Future directions

Since our initial foray into feature rating approaches (Crutch et al., 2012; Troche et al., 2014) several other researchers have used similar methods with great success. These methods are employed as they can be employed successfully to describe the semantic representation of both abstract and concrete concepts as we have successfully shown in our current work. Most recently, Binder and colleagues performed a feature rating approach with over 65 dimensions (Binder et al., 2016). They also were successful in using a feature rating approach to describe not only the representation of concrete concepts but abstract concepts as well.

The interesting work by Binder has added a key piece to our understanding of the normal functioning of the semantic system. We, however, remain interested in how the semantic space might change due to age or neurological disease (e.g., Parkinson's Disease, Corticobasal Degeneration, Semantic Dementia). Having older adults and persons with neurological disease perform a feature rating approach would be an excellent method for describing these differences. In this case, our CFR approach would likely be best with these groups due to the inherent difficulties in recruitment and limitations in time associated with them. Also with enough normative data with these groups, this approach could become a sensitive screening measure for neurological disease. We know that a variety of neurological disorders affect the semantic system differently. Therefore, the patterns in response might become an early indicator of a certain neurological disease. This would also bring us closer to an evaluation tool for abstract concept loss for which few options exist.

Another important question that remains unanswered specific to the CFR space is how our space would map onto the brain space. It would appear to map well onto the brain space with each of the three factors being part of high level cognitive networks. Our work, however, cannot speak to the relationship between these brain areas and our latent factors. A study which invokes the use of functional connectivity analyses would be required in order to describe the highly distributed networks of these high level cognitive processes.

If the CFR space does map onto the brain, then our CFR space could become a powerful computational model that would allow us to understand the way that concepts interact when certain brain areas are damaged. For example, Corticobasal degeneration (CBD) is a disorder which leads to lesioning of the parietal lobe with accompanying magnitude system deficits. This would be akin to lesioning a portion of the magnitude axis in of CFR space. By using the CFR space in this way, we would be able to understand better deficits in concept knowledge in a variety of disorders which would have huge implications for the assessment and treatment of conceptual knowledge.

## Ethics statement

This study was carried out in accordance with the recommendations of the University of Florida, Institutional Review Board with written informed consent from all subjects. All subjects gave written informed consent in accordance with the Declaration of Helsinki. The protocol was approved by the University of Florida, Institutional Review Board.

## Author contributions

All authors listed have made a substantial, direct and intellectual contribution to the work, and approved it for publication.

### Conflict of interest statement

The authors declare that the research was conducted in the absence of any commercial or financial relationships that could be construed as a potential conflict of interest.

## References

[B1] Aldenderfer M. S. Blashfield R. K. (1984). Cluster Analysis. Thousand Oaks, CA: Sage Publications.

[B2] Allen R. Hulme C. (2006). Speech and language processing mechanisms in verbal serial recall. J. Mem. Lang. 55, 64–88. 10.1016/j.jml.2006.02.002

[B3] Allman M. J. Meck W. H. (2012). Pathophysiological distortions in time perception and timed performance. Brain 135, 656–677. 10.1093/brain/awr210 21921020 PMC3491636

[B4] Altarriba J. Bauer L. Benvenuto C. (1999). Concreteness, context availability, and imageability ratings and word associations for abstract, concrete, and emotion words. Behav. Res. Methods 31, 578–602. 10.3758/BF03200738 10633977

[B5] Amodio D. M. Frith C. D. (2006). Meeting of minds: the medial frontal cortex and social cognition. Nat. Rev. Neurosci. 7, 268–277. 10.1038/nrn1884 16552413

[B6] Anderson T. W. Rubin H. (1956). Statistical inference in factor analysis. Proc. Berkeley Symp. Mathemat. Stat. Probab. 5, 111–150.

[B7] Andrews M. Vigliocco G. Vinson D. (2009). Integrating experiential and distributional data to learn semantic representations. Psychol. Rev. 116, 463–498. 10.1037/a0016261 19618982

[B8] Barsalou L. W. (1999). Perceptions of perceptual symbols. Behav. Brain Sci. 22, 637–660. 10.1017/S0140525X99532147 11301525

[B9] Barsalou L. W. (2008). Grounded cognition. Annu. Rev. Psychol. 59, 617–645. 10.1146/annurev.psych.59.103006.093639 17705682

[B10] Binder J. R. Conant L. L. Humphries C. J. Fernandino L. Simons S. B. Aguilar M.. (2016). Toward a brain-based componential semantic representation. Cogn. Neuropsychol. 33, 130–174. 10.1080/02643294.2016.1147426 27310469

[B11] Binder J. R. Westbury C. F. McKiernan K. A. Possing E. T. Medler D. A. (2005). Distinct brain systems for processing concrete and abstract concepts. J. Cogn. Neurosci. 17, 905–917. 10.1162/0898929054021102 16021798

[B12] Bleasdale F. A. (1987). Concreteness-dependent associative priming: separate lexical organization for concrete and abstract words. J. Exp. Psychol. Learn. Mem. Cogn. 13, 582–594. 10.1037/0278-7393.13.4.582

[B13] Bonner M. F. Vesely L. Price C. Anderson C. Richmond L. Farag C.. (2009). Reversal of the concreteness effect in semantic dementia. Cogn. Neuropsychol. 26, 568–579. 10.1080/02643290903512305 20183015 PMC2918518

[B14] Borghi A. M. Flumini A. Cimatti F. Marocco D. Scorolli C. Borghi A.. (2011). Manipulating objects and telling words: a study on concrete and abstract words acquisition. Front. Psychol. 2:15. 10.3389/fpsyg.2011.00015 21716582 PMC3110830

[B15] Breedin S. D. Saffran E. M. Coslett H. B. (1994). Reversal of the concreteness effect in a patient with semantic dementia. Cogn. Neuropsychol. 11, 617–660. 10.1080/02643299408251987

[B16] Brysbaert M. New B. (2009). Moving beyond Kučera and Francis: a critical evaluation of current word frequency norms and the introduction of a new and improved word frequency measure for American English. Behav. Res. Methods 41, 977–990. 10.3758/brm.41.4.977 19897807

[B17] Brysbaert M. Warriner A. B. Kuperman V. (2014). Concreteness ratings for 40 thousand generally known English word lemmas. Behav. Res. Methods 46, 904–911. 10.3758/s13428-013-0403-5 24142837

[B18] Buhrmester M. Kwang T. Gosling S. D. (2011). Amazon's mechanical turk. Perspect. Psychol. Sci. 6, 3–5. 10.1177/1745691610393980 26162106

[B19] Chao L. L. Martin A. (1999). Cortical regions associated with perceiving, naming, and knowing about colors. J. Cogn. Neurosci. 11, 25–35. 10.1162/089892999563229 9950712

[B20] Chao L. L. Martin A. (2000). Representation of manipulable man-made objects in the Dorsal stream. Neuroimage 12, 478–484. 10.1006/nimg.2000.0635 10988041

[B21] Chiarelli V. El Yagoubi R. Mondini S. Bisiacchi P. Semenza C. (2011). The syntactic and semantic processing of mass and count nouns: an ERP study. PLoS ONE 6:e25885. 10.1371/journal.pone.0025885 21998715 PMC3187832

[B22] Cicchetti D. V. (1994). Guidelines, Criteria, and Rules of Thumb for Evaluating Normed and Standardized Assessment Instruments in Psychology, Vol. 6, American Psychological Association, 284–290.

[B23] Cipolotti L. Warrington E. K. (1995). Semantic memory and reading abilities: a case report. J. Int. Neuropsychol. Soc. 1, 104–110. 10.1017/S1355617700000163 9375215

[B24] Coltheart M. (1981). The MRC psycholinguistic database. Q. J. Exp. Psychol. A Hum. Exp. Psychol. 33, 497–505. 10.1080/14640748108400805

[B25] Coltheart M. Patterson K. Marshall J. C. (1987). Deep dyslexia since 1980, in Deep Dyslexia, 2nd Edn, eds Coltheart M. Patterson K. Marshall J. C. (New York, NY, Routledge), 407–451.

[B26] Connell L. Lynott D. (2012). Strength of perceptual experience predicts word processing performance better than concreteness or imageability. Cognition 125, 452–465. 10.1016/j.cognition.2012.07.010 22935248

[B27] Connell L. Lynott D. (2013). Flexible and fast: linguistic shortcut affects both shallow and deep conceptual processing. Psychon. Bull. Rev. 20, 542–550. 10.3758/s13423-012-0368-x 23307559

[B28] Coull J. T. Cheng R. K. Meck W. H. (2011). Neuroanatomical and neurochemical substrates of timing. Neuropsychopharmacology 36, 3–25. 10.1038/npp.2010.113 20668434 PMC3055517

[B29] Cree G. S. McRae K. (2003). Analyzing the factors underlying the structure and computation of the meaning of chipmunk, cherry, chisel, cheese, and cello (and many other such concrete nouns). J. Exp. Psychol. Gen. 132:163. 10.1037/0096-3445.132.2.163 12825636

[B30] Crutch S. J. Jackson E. C. (2011). Contrasting graded effects of semantic similarity and association across the concreteness spectrum. Q. J. Exp. Psychol. 64, 1388–1408. 10.1080/17470218.2010.543285 21337284

[B31] Crutch S. J. Troche J. Reilly J. Ridgway G. R. (2013). Abstract conceptual feature ratings: the role of emotion, magnitude, and other cognitive domains in the organization of abstract conceptual knowledge. Front. Hum. Neurosci. 7:186. 10.3389/fnhum.2013.00186 23720617 PMC3662089

[B32] Crutch S. J. Warrington E. K. (2003). Spatial coding of semantic information: knowledge of country and city names depends on their geographical proximity. Brain 126, 1821–1829. 10.1093/brain/awg187 12821525

[B33] Crutch S. J. Warrington E. K. (2005). Abstract and concrete concepts have structurally different representational frameworks. Brain 128, 615–627. 10.1093/brain/awh349 15548554

[B34] Crutch S. J. Williams P. Ridgway G. R. Borgenicht L. (2012). The role of polarity in antonym and synonym conceptual knowledge: evidence from stroke aphasia and multidimensional ratings of abstract words. Neuropsychologia 50, 2636–2644. 10.1016/j.neuropsychologia.2012.07.015 22820631

[B35] Dolan R. J. Vuilleumier P. (2003). Amygdala automaticity in emotional processing. Ann. N. Y. Acad. Sci. 985, 348–355. 10.1111/j.1749-6632.2003.tb07093.x 12724170

[B36] Drury H. A. Van Essen D. C. Anderson C. H. Lee C. W. Coogan T. A. Lewis J. W. (1996). Computerized mappings of the cerebral cortex: a multiresolution flattening method and a surface-based coordinate system. J. Cogn. Neurosci. 8, 1–28. 10.1162/jocn.1996.8.1.1 11539144

[B37] Farah M. J. McClelland J. L. (1991). A computational model of semantic memory impairment: modality specificity and emergent category specificity. J. Exp. Psychol. Gen. 120, 339–357. 10.1037/0096-3445.120.4.339 1837294

[B38] Franklin S. Howard D. Patterson K. (1995). Abstract word anomia. Cogn. Neuropsychol. 12, 549–566. 10.1080/02643299508252007

[B39] Gallese V. Lakoff G. (2005). The Brain's concepts: the role of the Sensory-motor system in conceptual knowledge. Cogn. Neuropsychol. 22, 455–479. 10.1080/02643290442000310 21038261

[B40] Garcia A. Troche J. Cohen R. (2015). The importance of concreteness, similarity, and association for semantic knowledge. Clin. Neuropsychol. 29:310.

[B41] Garrard P. Lambon-Ralph M. A. Hodges J. R. Patterson K. (2001). Prototypicality, distinctiveness, and intercorrelation: analyses of the semantic attributes of living and nonliving concepts. Cogn. Neuropsychol. 18, 125–174. 10.1080/02643290125857 20945209

[B42] Gathercole V. C. (1985). “He has too much hard questions”: the acquisition of the linguistic mass-count distinction in much and many. J. Child Lang. 12, 395–415. 10.1017/S0305000900006504 4019610

[B43] Gilhooly K. Logie R. (1980). Age-of-acquisition, imagery, concreteness, familiarity, and ambiguity measures for 1,944 words. Behav. Res. Methods 12, 395–427. 10.3758/BF03201693

[B44] Gordon P. (1985). Evaluating the semantic categories hypothesis: the case of the count/mass distinction. Cognition 20, 209–242. 10.1016/0010-0277(85)90009-5 4064607

[B45] Hauk O. Johnsrude I. Pulvermüller F. (2004). Somatotopic representation of action words in human motor and premotor cortex. Neuron 41, 301–307. 10.1016/s0896-6273(03)00838-9 14741110

[B46] Howard M. W. Kahana M. J. (1999). Contextual variability and serial position effects in free recall. J. Exp. Psychol. Learn. Mem. Cogn. 25:923. 10.1037/0278-7393.25.4.923 10439501

[B47] Howard M. W. Kahana M. J. (2002). When does semantic similarity help episodic retrieval? J. Mem. Lang. 46, 85–98. 10.1006/jmla.2001.2798

[B48] Jones-Gotman M. Zatorre R. J. (1993). Odor recognition memory in humans: role of right temporal and orbitofrontal regions. Brain Cogn. 22, 182–198. 10.1006/brcg.1993.1033 8373572

[B49] Kaiser H. (1958). The varimax criterion for analytic rotation in factor analysis. Psychometrika 23, 187–200. 10.1007/BF02289233

[B50] Katz R. B. Goodglass H. (1990). Deep dysphasia: analysis of a rare form of repetition disorder. Brain Lang. 39, 153–185. 10.1016/0093-934X(90)90009-6 2207619

[B51] Klatzky R. L. Lederman S. J. Reed C. (1987). There's more to touch than meets the eye: the salience of object attributes for haptics with and without vision. J. Exp. Psychol. Gen. 116, 356–369. 10.1037/0096-3445.116.4.356

[B52] Kousta S. T. Vigliocco G. Vinson D. P. Andrews M. Del Campo E. (2011). The representation of abstract words: why emotion matters. J. Exp. Psychol. Gen. 140, 14–34. 10.1037/a0021446 21171803

[B53] Kousta S. T. Vinson D. P. Vigliocco G. (2009). Emotion words, regardless of polarity, have a processing advantage over neutral words. Cognition 112, 473–481. 10.1016/j.cognition.2009.06.007 19591976

[B54] Kulkarni R. Rothstein S. Treves A. (2013). A statistical investigation into the cross-linguistic distribution of mass and count nouns: Morphosyntactic and semantic perspectives. Biolinguistics 7, 132–168.

[B55] Lakoff G. Johnson M. (2008). Metaphors We Live By. Chicago, IL: University of Chicago Press.

[B56] Landauer T. K. Dumais S. T. (1997). A solution to Plato's problem: the latent semantic analysis theory of acquisition, induction, and representation of knowledge. Psychol. Rev. 104, 211 10.1037/0033-295X.104.2.211

[B57] Lederman S. J. Klatzky R. L. (1987). Hand movements: a window into haptic object recognition. Cogn. Psychol. 19, 342–368. 10.1016/0010-0285(87)90008-9 3608405

[B58] Lynott D. Connell L. (2013). Modality exclusivity norms for 400 nouns: the relationship between perceptual experience and surface word form. Behav. Res. Methods 45, 516–526. 10.3758/s13428-012-0267-0 23055172

[B59] Marshall J. Pring T. Chiat S. Robson J. (1996). Calling a salad a federation: an investigation of semantic jargon. Part 1—nouns. J. Neuroling. 9, 237–250. 10.1016/S0911-6044(97)82796-0

[B60] Martin A. (2007). The representation of object concepts in the brain. Annu. Rev. Psychol. 58, 25–45. 10.1146/annurev.psych.57.102904.190143 16968210

[B61] Martin N. Saffran E. M. (1992). A computational account of deep dysphasia: evidence from a single case study. Brain Lang. 43, 240–274. 10.1016/0093-934X(92)90130-7 1393522

[B62] Matell M. S. Meck W. H. (2000). Neuropsychological mechanisms of interval timing behavior. Bioessays 22, 94–103. 10.1002/(SICI)1521-1878(200001)22:1<94::AID-BIES14>3.0.CO;2-E 10649295

[B63] McRae K. Cree G. S. Seidenberg M. S. McNorgan C. (2005). Semantic feature production norms for a large set of living and nonliving things. Behav. Res. Methods 37, 547–559. 10.3758/bf03192726 16629288

[B64] Meck W. H. (2005). Neuropsychology of timing and time perception. Brain Cogn. 58, 1–8. 10.1016/j.bandc.2004.09.004 15878722

[B65] Meier B. P. Robinson M. D. (2004). Why the sunny side is up: associations between affect and vertical position. Psychol. Sci. 15, 243–247. 10.1111/j.0956-7976.2004.00659.x 15043641

[B66] Meteyard L. Cuadrado S. R. Bahrami B. Vigliocco G. (2012). Coming of age: a review of embodiment and the neuroscience of semantics. Cortex 48, 788–804. 10.1016/j.cortex.2010.11.002 21163473

[B67] Mishkin M. Ungerleider L. G. Macko K. A. (1983). Object vision and spatial vision: two cortical pathways. Trends Neurosci. 6, 414–417. 10.1016/0166-2236(83)90190-X

[B68] Molholm S. Ritter W. Javitt D. C. Foxe J. J. (2004). Multisensory visual–auditory object recognition in humans: a high-density electrical mapping study. Cereb. Cortex 14, 452–465. 10.1093/cercor/bhh007 15028649

[B69] Moll J. Zahn R. de Oliveira-Souza R. Krueger F. Grafman J. (2005). The neural basis of human moral cognition. Nat. Rev. Neurosci. 6, 799–809. 10.1038/nrn1768 16276356

[B70] Moscovitch M. Nadel L. Winocur G. Gilboa A. Rosenbaum R. S. (2006). The cognitive neuroscience of remote episodic, semantic and spatial memory. Curr. Opin. Neurobiol. 16, 179–190. 10.1016/j.conb.2006.03.013 16564688

[B71] Newcombe P. I. Campbell C. Siakaluk P. D. Pexman P. M. (2012). Effects of emotional and sensorimotor knowledge in semantic processing of concrete and Abstract Nouns. Front. Hum. Neurosci. 6:275. 10.3389/fnhum.2012.00275 23060778 PMC3465854

[B72] Ostergaard A. Davidoff J. (1985). Some effects of color on naming and recognition of objects. J. Exp. Psychol. Learn. Mem. Cogn. 11:579. 10.1037/0278-7393.11.3.579 3160817

[B73] Paivio A. (1991). Dual coding theory: retrospect and current status. Can. J. Psychol. Rev. Can. Psychol. 45, 255–287. 10.1037/h0084295

[B74] Paivio A. (2013). Dual coding theory, word abstractness, and emotion: a critical review of Kousta et al. (2011). J. Exp. Psychol. Gen. 142, 282–287. 10.1037/a0027004 23398183

[B75] Paivio A. Yuille J. C. Madigan S. A. (1968). Concreteness, imagery, and meaningfulness values for 925 nouns. J. Exp. Psychol. 76(1, Pt.2), 1–25. 10.1037/h0025327 5672258

[B76] Papagno C. Capasso R. Zerboni H. Miceli G. (2007). A reverse concreteness effect in a subject with semantic dementia. Brain and Lang. 103, 90–91. 10.1016/j.bandl.2007.07.059

[B77] Pecher D. Boot I. van Dantzig S. (2011). Abstract Concepts: Sensory-Motor Grounding, Metaphors, and Beyond. Available online at: http://hdl.handle.net/1765/30616

[B78] Reed C. L. Shoham S. Halgren E. (2004). Neural substrates of tactile object recognition: an fMRI study. Hum. Brain Mapp. 21, 236–246. 10.1002/hbm.10162 15038005 PMC6871926

[B79] Reilly J. Peelle J. E. Grossman M. (2007). A unitary semantics account of reverse concreteness effects in semantic dementia. Brain Lang. 103, 86–87. 10.1016/j.bandl.2007.07.057

[B80] Rock I. Victor J. (1964). Vision and touch: an experimentally created conflict between the two senses. Science 143, 594–596. 14080333 10.1126/science.143.3606.594

[B81] Roeltgen D. P. Sevush S. Heilman K. M. (1983). Phonological agraphia: writing by the lexical-semantic route. Neurology 33, 755–765. 10.1212/WNL.33.6.755 6682519

[B82] Rolls E. T. (2000). The orbitofrontal cortex and reward. Cereb. Cortex 10, 284–294. 10.1093/cercor/10.3.284 10731223

[B83] Royet J.-P. Zald D. Versace R. Costes N. Lavenne F. Koenig O.. (2000). Emotional responses to pleasant and unpleasant olfactory, visual, and auditory stimuli: a positron emission tomography study. J. Neurosci. 20, 7752–7759. 11027238 10.1523/JNEUROSCI.20-20-07752.2000 PMC6772882

[B84] Scalia F. Winans S. S. (1975). The differential projections of the olfactory bulb and accessory olfactory bulb in mammals. J. Compar. Neurol. 161, 31–55. 10.1002/cne.901610105 1133226

[B85] Schwanenflugel P. J. Harnishfeger K. K. Stowe R. W. (1988). Context availability and lexical decisions for abstract and concrete words. J. Mem. Lang. 27, 499–520. 10.1016/0749-596X(88)90022-8

[B86] Schwanenflugel P. J. Shoben E. J. (1983). Differential context effects in the comprehension of abstract and concrete verbal materials. J. Exp. Psychol. Learn. Mem. Cogn. 9, 82–102. 10.1037/0278-7393.9.1.82

[B87] Scorolli C. Jacquet P. O. Binkofski F. Nicoletti R. Tessari A. Borghi A. M. (2012). Abstract and concrete phrases processing differentially modulates cortico-spinal excitability. Brain Res. 1488, 60–71. 10.1016/j.brainres.2012.10.004 23044471

[B88] Small D. M. Jones-Gotman M. Zatorre R. J. Petrides M. Evans A. C. (1997). Flavor processing: more than the sum of its parts. Neuroreport 8, 3913–3917. 10.1097/00001756-199712220-00014 9462465

[B89] Small D. M. Zald D. H. Jones-Gotman M. Zatorre R. J. Pardo J. V. Frey S.. (1999). Human cortical gustatory areas: a review of functional neuroimaging data. Neuroreport 10, 7–13. 10.1097/00001756-199901180-00002 10094124

[B90] Stein B. E. London N. Wilkinson L. K. Price D. D. (1996). Enhancement of perceived visual intensity by auditory stimuli: a psychophysical analysis. J. Cogn. Neurosci. 8, 497–506. 10.1162/jocn.1996.8.6.497 23961981

[B91] Stuss D. T. Shallice T. Alexander M. P. Picton T. W. (1995). A multidisciplinary approach to anterior attentional functionsa. Ann. N. Y. Acad. Sci. 769, 191–212. 10.1111/j.1749-6632.1995.tb38140.x 8595026

[B92] Tanaka J. W. Presnell L. M. (1999). Color diagnosticity in object recognition. Percept. Psychophys. 61, 1140–1153. 10.3758/BF03207619 10497433

[B93] Toglia M. P. Battig W. F. (1978). Handbook of Semantic Word Norms. Oxford: Lawrence Erlbaum.

[B94] Troche J. Crutch S. J. Reilly J. (2014). Clustering, hierarchical organization, and the topography of abstract and concrete nouns. Front. Psychol. 5:360. 10.3389/fpsyg.2014.00360 24808876 PMC4009417

[B95] Troche J. Reilly J. (2016). Eye tracking & pupillometry as a means to determine the validity of the multidimensional semantic space, in Paper Presented at the American Speech-Language-Hearing Association Convention, (Philadelphia, PA).

[B96] Tyler L. K. Moss H. E. Durrant-Peatfield M. R. Levy J. P. (2000). Conceptual structure and the structure of concepts: a distributed account of category-specific deficits. Brain Lang. 75, 195–231. 10.1006/brln.2000.2353 11049666

[B97] Van Essen D. C. Anderson C. H. Felleman D. J. (1992). Information processing in the primate visual system: an integrated systems perspective. Science 255, 419–423. 1734518 10.1126/science.1734518

[B98] Van Overwalle F. Baetens K. (2009). Understanding others' actions and goals by mirror and mentalizing systems: a meta-analysis. Neuroimage 48, 564–584. 10.1016/j.neuroimage.2009.06.009 19524046

[B99] Vigliocco G. Kousta S.-T. Della Rosa P. A. Vinson D. P. Tettamanti M. Devlin J. T.. (2013). The neural representation of abstract words: the role of emotion. Cereb. Cortex 24, 1767–1777. 10.1093/cercor/bht025 23408565

[B100] Walker I. Hulme C. (1999). Concrete words are easier to recall than abstract words: evidence for a semantic contribution to short-term serial recall. J. Exp. Psychol. Learn. Mem. Cogn. 25, 1256–1271.

[B101] Walsh V. (2003). A theory of magnitude: common cortical metrics of time, space and quantity. Trends Cogn. Sci. 7, 483–488. 10.1016/j.tics.2003.09.002 14585444

[B102] Ward J. H. Jr. (1963). Hierarchical grouping to optimize an objective function. J. Am. Stat. Assoc. 58, 236–244. 10.1080/01621459.1963.10500845

[B103] Warriner A. B. Kuperman V. Brysbaert M. (2013). Norms of valence, arousal, and dominance for 13,915 English lemmas. Behav. Res. Methods 45, 1191–1207. 10.3758/s13428-012-0314-x 23404613

[B104] Warrington E. K. (1975). The selective impairment of semantic memory. Q. J. Exp. Psychol. 27, 635–657. 10.1080/14640747508400525 1197619

[B105] Warrington E. Shallice T. (1984). Category specific semantic impairments. Brain 107, 829–853. 10.1093/brain/107.3.829 6206910

[B106] White L. E. (1965). Olfactory bulb projections of the rat. Anat. Rec. 152, 465–479. 10.1002/ar.1091520406

[B107] Wiemer-Hastings K. Xu X. (2005). Content differences for abstract and concrete concepts. Cogn. Sci. 29, 719–736. 10.1207/s15516709cog0000_33 21702791

[B108] Wurm L. H. Legge G. E. Isenberg L. M. Luebker A. (1993). Color improves object recognition in normal and low vision. J. Exp. Psychol. Hum. Percept. Perform. 19:899. 10.1037/0096-1523.19.4.899 8409865

[B109] Zahn R. Moll J. Iyengar V. Huey E. D. Tierney M. Krueger F.. (2009). Social conceptual impairments in frontotemporal lobar degeneration with right anterior temporal hypometabolism. Brain 132, 604–616. 10.1093/brain/awn343 19153155 PMC2724922

[B110] Zald D. H. Lee J. T. Fluegel K. W. Pardo J. V. (1998). Aversive gustatory stimulation activates limbic circuits in humans. Brain 121, 1143–1154. 10.1093/brain/121.6.1143 9648549

[B111] Zanini C. Benavides-Varela S. Lorusso R. Franzon F. (2016). Mass is more: the conceiving of (un)countability and its encoding into language in 5-year-old-children. Psychon. Bull. Rev. 24, 1330–1340. 10.3758/s13423-016-1187-2 27812960

[B112] Zaromb F. M. Howard M. W. Dolan E. D. Sirotin Y. B. Tully M. Wingfield A.. (2006). Temporal associations and prior-list intrusions in free recall. J. Exp. Psychol. Learn. Mem. Cogn. 32:792. 10.1037/0278-7393.32.4.792 16822147

[B113] Zatorre R. J. Jones-Gotman M. Evans A. C. Meyer E. (1992). Functional localization and lateralization of human olfactory cortex. Nature 360, 339–340 10.1038/360339a0 1448149

[B114] Zwaan R. A. Yaxley R. H. (2003). Spatial iconicity affects semantic relatedness judgments. Psychon. Bull. Rev. 10, 954–958. 10.3758/BF03196557 15000544

